# An inflammatory response-related gene signature can predict the prognosis and impact the immune infiltration of multiple myeloma

**DOI:** 10.1007/s10238-023-01277-w

**Published:** 2024-01-27

**Authors:** Qian Zhao, Feng Li, Jing Li, Yuan Xia, Jing Wang, Lijuan Chen

**Affiliations:** 1 Department of Hematology, Jiangsu Province Hospital, The First Affiliated Hospital of Nanjing Medical University, Nanjing, 210003 China; 2 Department of Hematology, Jinling Hospital, Nanjing Medical University, Nanjing, 210002 China

**Keywords:** Inflammatory response-related genes, Multiple myeloma, Prognostic, CD81, Immune infiltration

## Abstract

Multiple myeloma (MM) is a highly heterogeneous and incurable disease. Inflammation plays a vital role in cancer genesis and progression. However, the relationship between inflammatory response-related genes (IRRGs) and the prognosis of MM patients remains unknown. We constructed a IRRGs prognosis model by least absolute shrinkage and selection operator regression analysis. Moreover, clinical multivariate regression was performed to identify clinical implications. Gene set enrichment analysis was implemented to conduct its biological properties. CIBERSORT deconvolution algorithm was utilized to calculate the immune cell infiltration in different risk groups. The flow cytometry was utilized to perform protein expression of prognostic gene. A Six-IRRGs (VCAM1, RGS1, KIT, CD81, BLNK, and BIRC3) prognostic risk model was successfully constructed and validated. The risk model was an independent predictor for overall survival. Enrichment analysis revealed autophagy and PI3K–Akt signaling pathways were enriched in the high-risk group. Furthermore, we found CD81 widely impacted on the infiltration of immune cells, especially on monocytes and macrophages2. At last, the role of CD81 in MM was confirmed to be an adverse prognostic factor in clinical. Our study explores the potential application value of IRRGs in MM. These findings may provide new insights into the treatment for MM patients.

**Supplementary Information:**

The online version contains supplementary material available at 10.1007/s10238-023-01277-w.

## Introduction

Multiple myeloma (MM) is the second most common hematological malignancy characterized by malignant terminally differentiated plasma cells [1]. During the last decades, advances in anti-myeloma therapeutics, including proteasome inhibitors, immunomodulatory drugs, and anti-CD38 monoclonal antibodies have considerably improved the treatment outcome in myeloma [2]. Despite these improvements, most patients still face relapse and/or drug resistance. Thus, exploring a new mechanism of MM and searching for novel targets are urgently needed.

Inflammation predisposes to the development of cancer and promotes all stages of tumorigenesis [3]. According to early research reported by Lindqvist, a personal history of all infections combined was associated with a significantly increased risk of MM [4]. At present, a growing number of evidence indicates that inflammation plays a crucial role in the recurrence and drug resistance of MM. Because inflammatory immune cells not just participated in the formation of the tumor microenvironment (TME), but involved in tumor survival and immune modulation [5, 6]. Recently, single-cell transcriptomic datasets of MM tracking stromal inflammation in individuals over time revealed that successful antitumor induction therapy is unable to revert bone marrow inflammation [6]. Relapsed/refractory multiple myeloma (RRMM) cells can also shape an immune suppressive bone marrow microenvironment by up-regulation of inflammatory cytokines [7]. Furthermore, clinical and laboratory research suggested that use of anti-inflammatory agents is a promising approach for cancer prevention and treatment [8]. Currently, several studies have demonstrated that inflammatory response-related genes (IRRGs) could be used to predict the prognosis of various cancers, such as pancreatic ductal adenocarcinoma, hepatocellular carcinoma and transitional bladder cancer [9–11]. However, the relationship between IRRGs and the prognosis of MM patients remains unclear.

In this study, we collected the transcriptional expression and matched clinical data of patients with MM from Gene Expression Omnibus (GEO) and The Cancer Genome Atlas (TCGA) databases. Subsequently, we constructed and validated a risk model with differentially expressed genes (DEGs) related to inflammatory response. Then, the potential mechanism was explored in high-risk group by gene set enrichment analysis (GSEA) and the correlation analysis between IRRGs and infiltration of immune cells was carried out. Finally, we identified CD81 as one of the IRRGs has impacted the immune status extensively and displayed a bad prognosis as well.

## Methods

### Data collection for the identification of DEGs related to inflammatory response

**Fig. 1:**
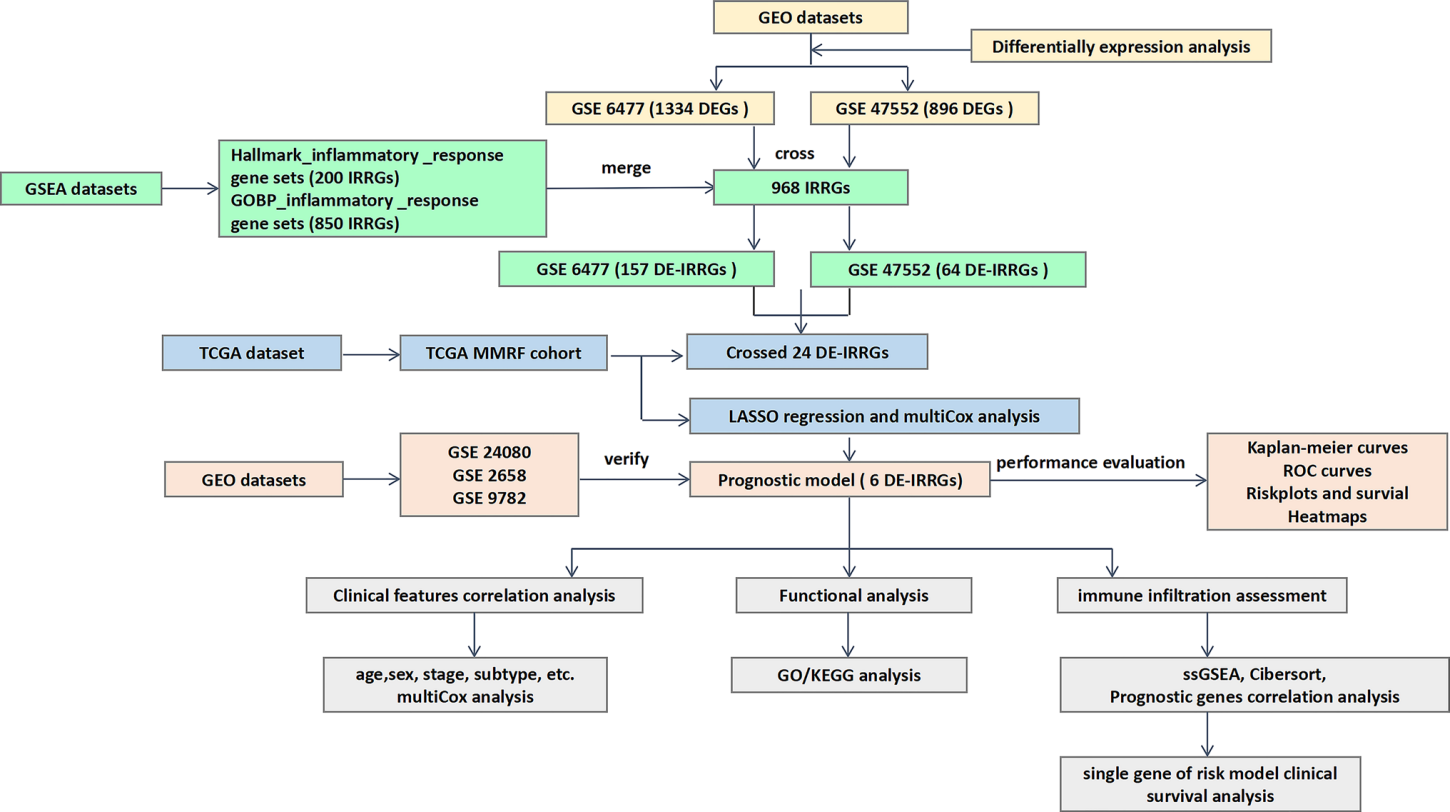
Research diagram of the informatics procedure

Figure 1 shows a flowchart of the procedure of this study. Our study obtained the data of MM samples from six public datasets. First, we obtained two datasets from the GEO database (https://www.ncbi.nlm.nih.gov/geo/). These are 84 samples from the GSE6477 dataset (69 with MM and 15 with normal person) and 46 samples from the GSE47552 dataset (41 with MM and 5 with normal person). “Limma” R package was used to screened DEGs between MM and normal person in these two GSE cohorts respectively, which were defined as those with a false discovery rate < 0.05 and a |fold change|> 1. Then, 200 IRRGs were acquired from the “Hallmark inflammatory response” and 850 IRRGs from the “GOBP inflammatory response” gene sets in the Molecular Signature Database (http://gsea-msigdb.org). We merged them and get a new 968 IRRGs set. Besides, we took the intersection of the above two DEGs sets and the 968 IRRGs set to obtain differentially expressed-IRRGs (DE-IRRGs), respectively. Finally, we identified the crossed DE-IRRGs by intersecting again.

### Data collection for the construction and validation of a prognostic DE-IRRGs signature

The least absolute shrinkage and selection operator (LASSO) regression analysis was used to select and shrink variables with “glmnet” R package to construct a DE-IRRGs prognostic model. After the shrinkage process, variables with a regression coefficient equal to zero are excluded from the model. A tuning parameter lambda (λ) controls the amount of shrinkage, with increased shrinkage for higher λ values. An optimal λ was chosen when the partial likelihood deviance reached its lowest, which was based on the tenfold cross-validation. The risk scores of patients were calculated according to the expression level of each inflammatory response-related gene and its corresponding regression coefficient. The formula was established as follows:

$${\text{risk}}\;{\text{score}} = e^{{sum(each\; \, gene^{\prime } s \, \;\exp ression\; \times \;corresponding \, \;coeffcient)}} .$$

We selected TCGA-Multiple Myeloma Research Foundation (MMRF) cohort (https://portal.gdc.cancer.gov/repository) as a training set and it contains a large number of 859 patients with MM. The other three datasets from GEO database were used as validation sets to validate reliability of the prognostic signature. These are 559 newly diagnosed samples from the GSE24080 dataset, 559 newly diagnosed samples from the GSE2658 dataset and 264 relapsed samples from the GSE9782. According to the median risk score, patients were divided into high- and low-risk groups. We created a Kaplan–Meier curve to clarify the correlation between risk score and patient survival index, and visualized the risk plot, survival status, and heat map of four datasets through related R packages to further verify the accuracy of the predictive model. The R package time-dependent receiver operating characteristic (ROC) was used to predict MM patient survival for 1, 2, and 3-years. The area under the curve (AUC) value represents the accuracy of prediction.

Based on the risk model, the difference analysis of clinical futures between different risk groups was carried out, and a box plot was drawn. Multivariate Cox regression was used to verify the independence of the predictive model and analyze whether the risk score could still be used as an independent predictor for the patient’s survival under the case of multifactorial clinical characteristics (age, gender, clinical stage and lactic dehydrogenase, etc.). A *P* value < 0.05 was considered statistically significant.

### Gene set enrichment analysis and immune infiltration estimations

To explore the biological functions and pathways of the gene signatures in the TCGA MMRF cohort, GSEA was utilized to conduct Gene Ontology (GO) and Kyoto Encyclopedia of Genes and Genomes (KEGG) analyses with “org.Hs.eg.db” R package between up-regulated genes and down-regulated genes in the high-risk group. *P* value was adjusted by BH method. The infiltration of 28 immune cells were displayed as a heat map in each sample, calculated by single-sample gene set enrichment analysis (ssGSEA) with the “GSVA”R package.

In addition, to assess the composition of different immune cell types between different risk groups, we also used the CIBERSORT deconvolution algorithm to obtain matrix data for the proportion of 22 immune cells per tumor sample from RNA-sequencing data. We further visualized matrix-based data filtered by *P* < 0.05 with the violin chart. We also performed correlation analysis between different immune cells and visualized the corresponding results in the correlation matrix plot of immune cells.

### Correlation analysis between prognostic IRRGs and immune infiltration

In order to clarify the relationship between prognostic IRRGs and immune infiltration, Spearman correlation analysis was used to analyze the correlation between variables with “limma” and “reshape2” R package. We further visualized the relationship between variables using a heat map and lollipop charts.

### Verification of the protein expression value of prognostic gene in clinical practice by flow cytometry

A total of 178 patients with initial MM treated between 2014 and 2019 were collected from the Jinling Hospital. This study was approved by the Clinical Research Ethics Committee of Jinling Hospital, Nanjing, China. Written informed consent was waived by the ethics committee. All the patients had available follow-up data and flow cytometry data. Erythrocyte-lysed whole-bone marrow (BM) samples were stained using a direct immunofluorescence technique, with four-color monoclonal antibody combinations. Fresh BM (2 mL) was treated with EDTA to prevent coagulation, and fluorescein isothiocyanate (FITC)/ phycoerythrin (PE)/peridinin-chlorophyll-protein (Percp)/allophycocyanin (APC) was used for cell labeling, as follows: CD138/CD38/CD19/CD45; CD38/CD45/CD20/CD28; CD38/CD45/CD200/CD33; CD38/CD45/CD117/CD56; CD38/CD45/cyKappa/cyLambda; and CD38/CD45/CD27/CD81. CD81 is one of the IRRGs in our risk model. The protein expression of CD81 was considered positive when 20% or more of tumor plasma cells with showing antigen expression. Data were acquired on a MACS Quant™ (Miltenyi, Germany), and MACS Quantify™ software was used for the analysis. The baseline characteristics of the patients according to CD81 expression are shown in Table 1. The categorical variables were compared by the χ^2^ test. Progression-free survival (PFS) was defined as the time from the initial therapy to the first disease progression or death. Overall survival (OS) was recorded from the date of diagnosis until the date of death or last follow-up. The PFS and OS were calculated using the Kaplan–Meier method and were compared for different subgroups using the log-rank test. A *P* value < 0.05 was considered statistically significant. Analyses were performed using SPSS version 19.0 for Windows (IBM, Armonk, NK).

**Table 1:** Clinical characteristics associated with CD81 expression in 178 cases of multiple myeloma

Characteristic	CD81 positive [cases (%)]	CD81 negative [cases (%)]	*P* value
Gender			0.400
Female (%)	44 (40.7)	33 (47.1)	
Male (%)	64 (59.3)	37 (52.9)	
Age (years), median	60.5	59.5	0.530
Hemoglobin (g/L), median	91	86	0.123
Subtype			0.161
IgG	39 (36.1)	41 (58.6)	
IgA	28 (25.9)	11 (15.7)	
IgD	7 (6.5)	3 (4.3)	
IgE	1 (0.9)	0 (0)	
κ light chain	15 (13.9)	6 (8.6)	
λ light chain	16 (14.8)	8 (11.4)	
Non secretory	2 (1.9)	1 (1.4)	
DS stage			0.278
I	7 (6.5)	1 (1.4)	
II	8 (7.4)	6 (8.6)	
III	93 (86.1)	63 (90.0)	
ISS stage			0.191
I	16 (14.8)	18 (25.7)	
II	30 (27.8)	16 (22.9)	
III	62 (57.4)	36 (51.4)	
Cytogenetics	101	66	
13q-	44 (43.6)	27 (40.9)	0.734
1q21	49 (48.5)	28 (52.4)	0.440
t(4;14)	16 (15.8)	18 (27.3)	0.073
t(11;14)	17 (16.8)	11 (16.7)	0.798
t(14;16)	2 (1.9)	0	—
P53	3 (3.0)	4 (6.1)	0.436
Creatinine (umol/L)			0.341
< 176	78 (72.2)	55 (78.6)	
≥ 176	30 (27.8)	15 (21.4)	
Serum calcium			0.152
Normal	85 (78.7)	61 (87.1)	
Hypercalcemia	23 (21.3)	9 (12.9)	
Bone lesion			0.396
Yes	89 (82.4)	61 (84.3)	
No	19 (17.6)	9 (15.7)	
β2 microglobulin (mg/L)			0.054
< 5.5	52 (48.1)	44 (62.9)	
≥ 5.5	56 (51.9)	26 (39.2)	
LDH			0.584
Normal	87 (80.6)	54 (77.1)	
Abnormal	21 (19.4)	16 (22.9)	
Ratio of involved to uninvolved sFLC			0.149
< 100	78 (76.5)	54 (85.7)	
≥ 100	24 (23.5)	9 (14.3)	
Extramedullary infiltration			0.696
Yes	83 (76.9)	52 (74.3)	
No	25 (23.1)	18 (25.7)	

IgG: immunoglobulin G, IgA: immunoglobulin A, IgD: immunoglobulin D, IgE: immunoglobulin E, DS: Durie–Salmon, ISS: international staging system, LDH: lactate dehydrogenase, sFLC: serum free light chain

## Results

### Identification of DEGs related to inflammatory response

**Fig. 2:**
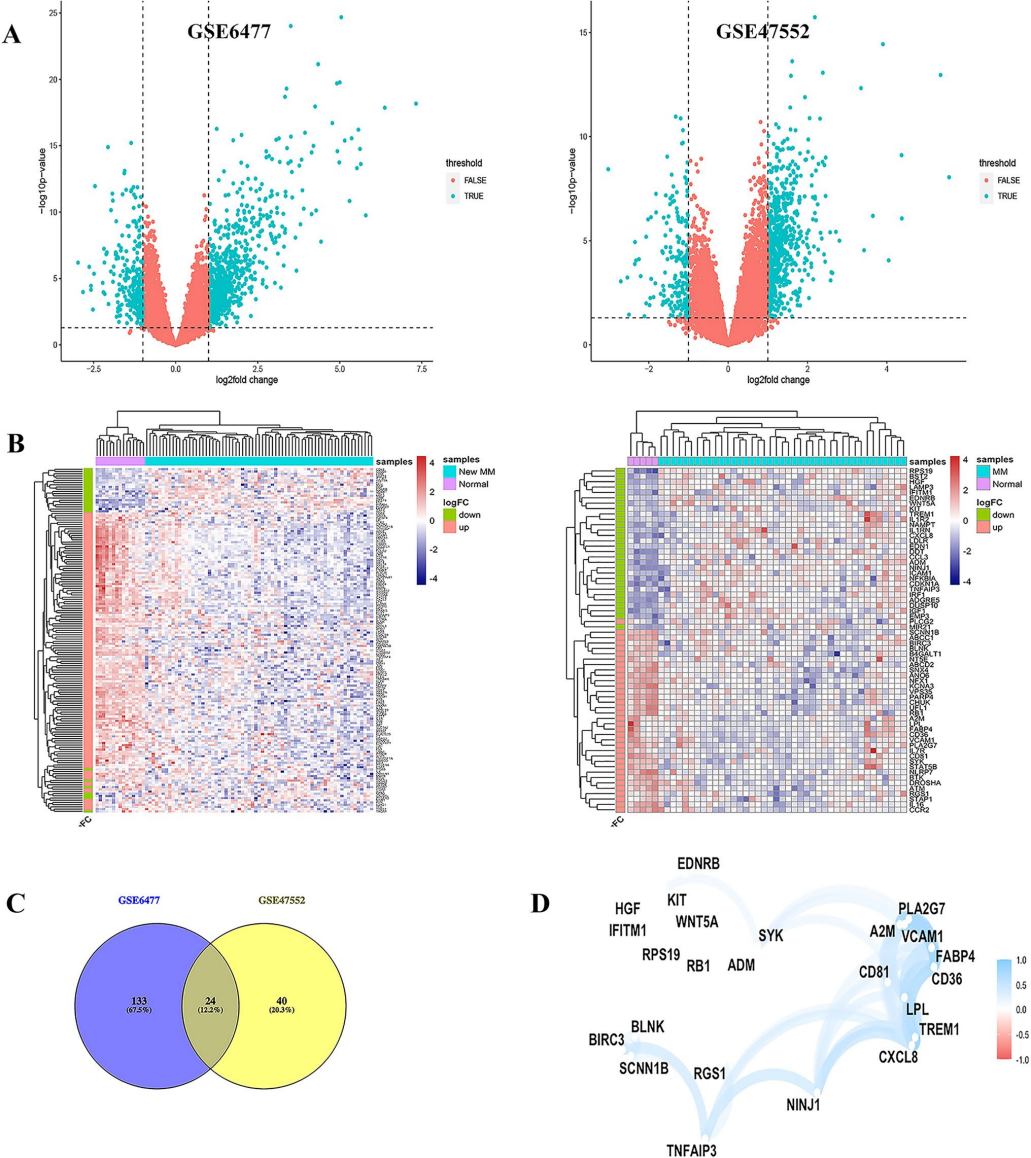
Identification of differentially expressed genes (DEGs) in two cohort profile datasets (GSE6477 and GSE47552). **A** Respective volcano plot of the two datasets. Green plots represent genes with [logFC] > 1 and p < 0.05. Red plots represent the remaining genes with no significant difference. **B** Heat map of the up- and down-regulated DE-IRRGs in the two datasets. **C** 24 crossed DE-IRRGs in the two datasets. **D** The correlation network of candidate DE-IRRGs

We identified 1334 and 896 DEGs from GSE6477 and GSE47552, respectively (Fig. 2A). There were 850 and 200 protein coding genes contained in the “GOBP inflammatory response” and “Hallmark inflammatory response” gene sets, respectively (Supplementary Tables S1 and S2). After removing the overlapped genes, a total of 968 IRRGs were obtained for further analysis. By extracting common DE-IRRGs, we obtained 157 and 64 shared DE-IRRGs in GSE6477 and GSE47552, respectively (Supplementary Tables S3 and S4). Figure 2B shows these genes depicted as a heat map. These genes were well clustered between patients with MM and healthy controls. In addition, by intersecting the DE-IRRGs of the 2 datasets, we obtained 24 crossed DE-IRRGs (Fig. 2C and Tables S5). Besides, Fig. 2D represents the interactions of these signatures.

### Construction of IRRGs prognostic model in the TCGA MMRF Cohort

**Fig. 3:**
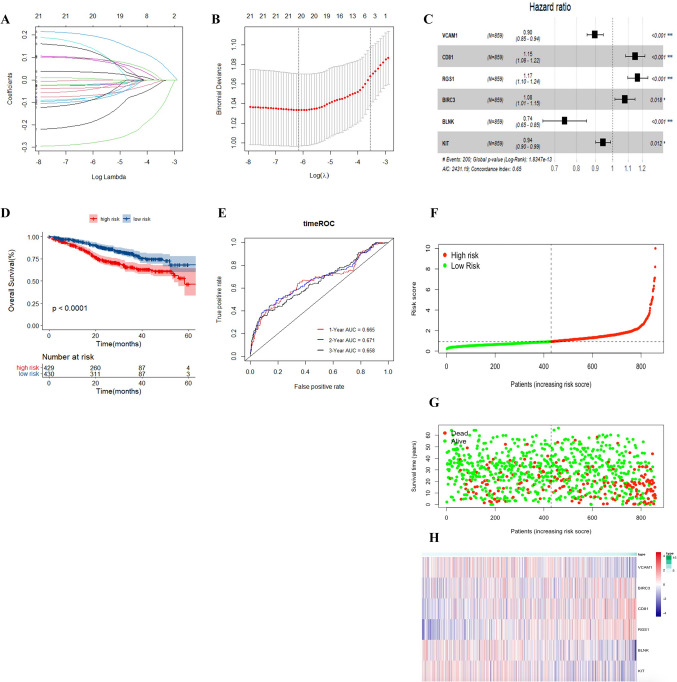
Prognostic analysis of the 6-IRRGs risk model in the TCGA cohort. **A** LASSO coefficient expression profiles of 24 candidate genes. **B** The penalty parameter (λ) in the LASSO model was selected through 10 cross-validation. **C** The forest plot of hazard ratios for by six IRRGs by multivariate Cox regression analysis. **D** Kaplan–Meier curves for OS of patients in the high- and low-risk groups of TCGA. **E** AUC time-dependent ROC curves for OS in TCGA. **F** The median value and distribution of the risk scores. **G** The distribution of OS status between the risk groups. **H** Expression profile of signature genes between the risk groups

PLAZG7 was excluded from this analysis because it is not present in TCGA MMRF cohort. The expression profiles of the above 23 genes were analyzed by LASSO regression analysis (Fig. 3A, B). A prognostic model consisting of six IRRGs (VCAM1, RGS1, KIT, CD81, BLNK, and BIRC3) was established based on the optimal value of lambda and multi-Cox regression model (Fig. 3C). The formula,

$${\text{risk}}\;{\text{score}} = e^{{\left( { - 0.1096 \, *{\text{ VCAM}}1 + \, 0.0750 \, *{\text{ BIRC}}3 + 0.1529 \, *{\text{ RGS}}1 \, + \, 0.1382 \, *{\text{ CD}}81 - 0.2965 \, *{\text{ BLNK }} - 0.0597 \, *{\text{ KIT}}} \right)}} ,$$

was used to compute the risk score of each patient.

We then divided patients in the TCGA MMRF cohort into high-risk and low-risk groups according to the median risk score. The Kaplan–Meier curve showed that patients in the high-risk group had significantly worse OS compared with those in the low-risk group (Fig. 3D, *P* < 0.0001). Next, to check the prediction performance of the risk score for patients with MM, time-dependent ROC analysis was used, and the AUC at 1, 2, and 3 years was 0.665, 0.671, and 0.658, respectively (Fig. 3E). Furthermore, the risk curve graph and survival state graph show the distribution of risk scores and the overall survival of each sample (Figs. 3F, G). The scatter chart indicated that patients in the high-risk group were more likely to die earlier than those in the low-risk group. The heat map shows the different distribution of the six IRRGs screened for the predictive model between the high- and low-risk groups (Figs. 3H).

### Validation of IRRGs prognostic model in the GEO Cohort

**Fig. 4:**
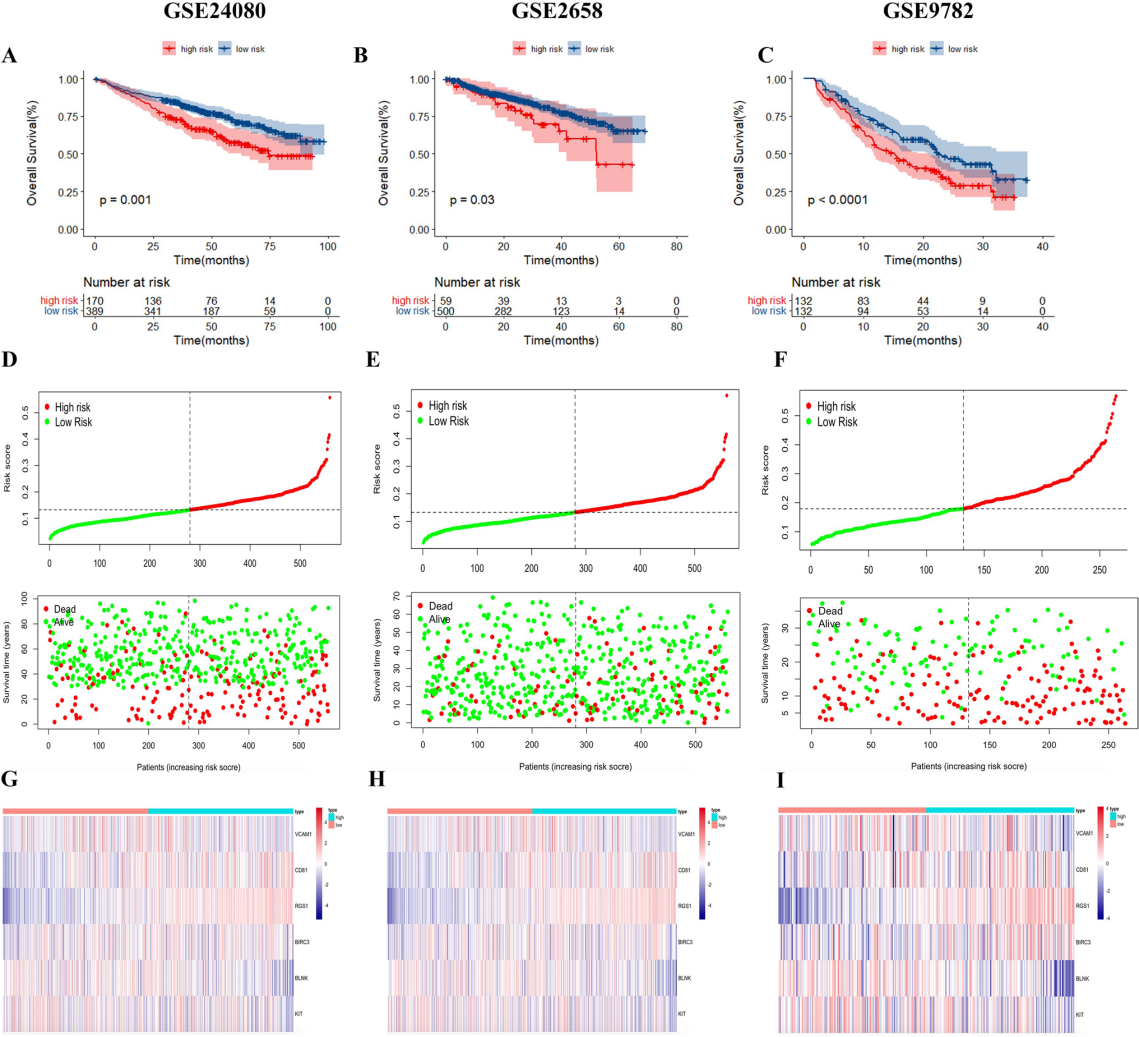
Role of the risk score in overall survival. **A** Kaplan–Meier curves for OS of patients in the high- and low-risk groups of GSE24080 cohort. **B** Kaplan–Meier curves for OS of patients in the high- and low-risk groups of GSE2658 cohort. **C** Kaplan–Meier curves for OS of patients in the high- and low-risk groups of GSE9782 cohort. **D** and **F** The distributions of risk score, survival status between the high- and low-risk groups of GSE24080 **D**, GSE2658 **E** and GSE9782 **F** cohorts. (4G-I) Expression profile of signature genes between the risk groups of GSE24080 **G**, GSE2658 **H** and GSE9782 **I** cohorts

To verify the reliability and stability of the prognostic model constructed from the TCGA MMRF cohort, patients in the GEO cohort (GSE24080, GSE2658 and GSE9782) were also categorized into high-risk and low-risk groups according to the median value from the prognostic model. Similarly, whether initial MM or RRMM, the results of the Kaplan–Meier plot show that patients with high-risk score had a lower survival probability than those with a low-risk score (Fig. 4A–C). Similar to the results obtained from the TCGA MMRF cohort, patients with a high-risk score had a higher death probability than those with a low-risk score (Fig. 4D–F), and expression levels of the six IRRGs were significantly different between high- and low-risk groups (Fig. 4G–I).

### Clinical implication of the risk score

**Fig. 5:**
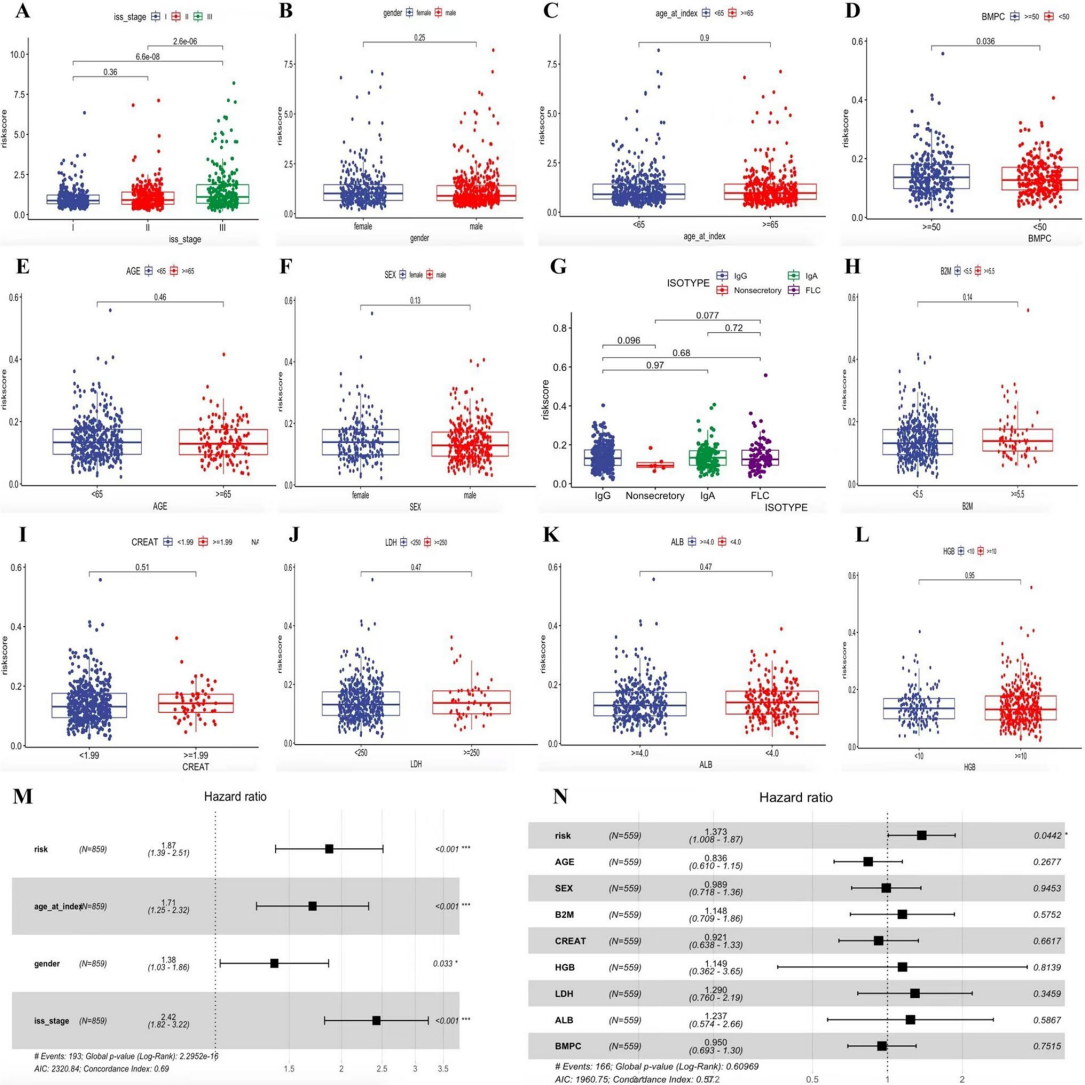
Risk score in different groups divided by clinical characteristics in TCGA. **A** international staging system (ISS) stage, **B** gender and **C** Age; The risk score in different groups divided by clinical characteristics in GSE24080. **D** bone marrow plasma cell infiltration (BMPC), **E** age, **F** gender, **G** isotype, **H** β2 microglobulin, **I** creatinine, **J** lactate dehydrogenase, **K** albumin and **L** hemoglobin. **M** multivariate Cox regression analysis in TCGA. **N** multivariate Cox regression analysis in GSE24080

To elucidate the clinical implication of the risk model, clinical variables such as age, gender, and international staging system (ISS) variables were enrolled into the correlation analysis. Box plot showed that patients with ISS II-III stage are more likely have a high-risk score (Figs. 5A) in the TCGA MMRF cohort. However, the relationship between gender, age variable and the risk score was not significant (Fig. 5B, C). In addition, the same analysis in the GSE24080 cohort confirmed that the risk score was definitely higher in bone marrow plasma cell infiltration (BMPC) ≥ 50% compared with BMPC** < **50% (There was no data about ISS stage of MM in the GSE24080 cohort) (Fig. 5D). Likewise, there was no significant correlation between age, gender, isotype, β2 microglobulin, creatinine, lactate dehydrogenase (LDH), albumin, hemoglobin and risk score (Fig. 5E–L). Finally, multivariate Cox regression analysis was performed and indicate that the risk score is an independent prognostic factor for MM patients both in the TCGA MMRF cohort and GSE24080 cohort (Fig. 5M, N).

### Enrichment analysis and immune infiltration assessment

**Fig. 6:**
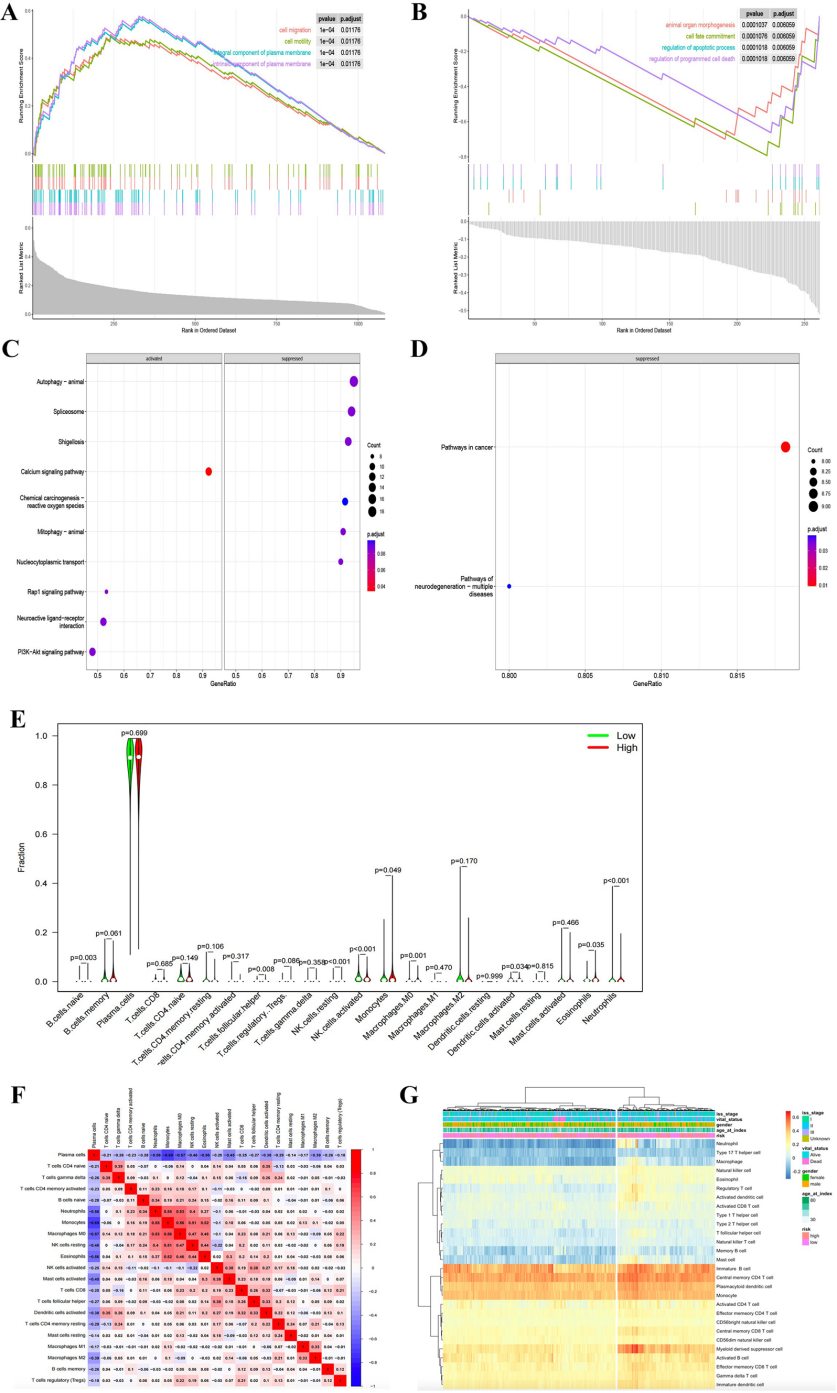
GSEA enrichment and immune infiltration assessment analyses. (**A** and **B**) GO enrichment analysis for up-regulated genes and down-regulated genes in the high-risk group. Based on biological features. (**C** and **D**) KEGG enrichment analysis for up-regulated genes and down-regulated genes in the high-risk group. **E** Violin plot of the infiltration abundance of 22 immune cell types in patients with high and low-risk score in TCGA. **F** The correlation analysis of 22 immune cell in TCGA (red: positive correlation; blue: negative correlation; the darker the color of the block, the correlation coefficient is closer to 1 or -1). **G** An overview heat map of 22 different immune cells

The GSEA was used to perform GO function and KEGG pathway enrichment analyses between up-regulated genes and down-regulated genes in the high-risk group of TCGA MMRF cohort. A number of biological functions were enriched in up-regulated genes, including cell migration, cell motility, integral component of plasma membrane and intrinsic component of plasma membrane (Fig. 6A). The functions of down-regulated genes were enriched in animal organ morphogenesis, cell fate commitment, regulation of apoptotic process and regulation of programmed cell death (Fig. 6B). Meanwhile, we found the KEGG pathways of up-regulated genes were enriched in autophagy, spliceosome signaling pathway, which were suppressed (Fig. 6C) and in calcium signaling pathway, neuroactive ligand-receptor interaction and PI3K-Akt signaling pathway, which were activated. However, the KEGG pathways of down-regulated genes were mainly enriched in pathways in cancer (Fig. 6D).

Based on the CIBERSORT deconvolution algorithm, we compared the proportion of 22 types of immune cells between high-and low-risk groups. As shown in Fig. 6E, B cells memory, NK cells activated, macrophages M0, Dendritic cells activated, neutrophils were significantly higher in the low-risk group, while B cells naïve, T cells follicular helper, NK cells resting, monocytes and Eosinophils were more likely higher in the high-risk group. In addition, according to the correlation coefficient of immune cells, plasma cells were significantly negatively correlated with neutrophils, monocytes, macrophages M0, eosinophils and NK cells resting. However, neutrophils, monocytes, macrophages M0, eosinophils and NK cells resting were positively correlated with each other (Fig. 6F). Based on several different algorithms, the heat map demonstrated the heterogeneity in the immune landscape among the subgroups (Fig. 6G).

### Relationship of prognostic IRRGs and immune infiltration

**Fig. 7:**
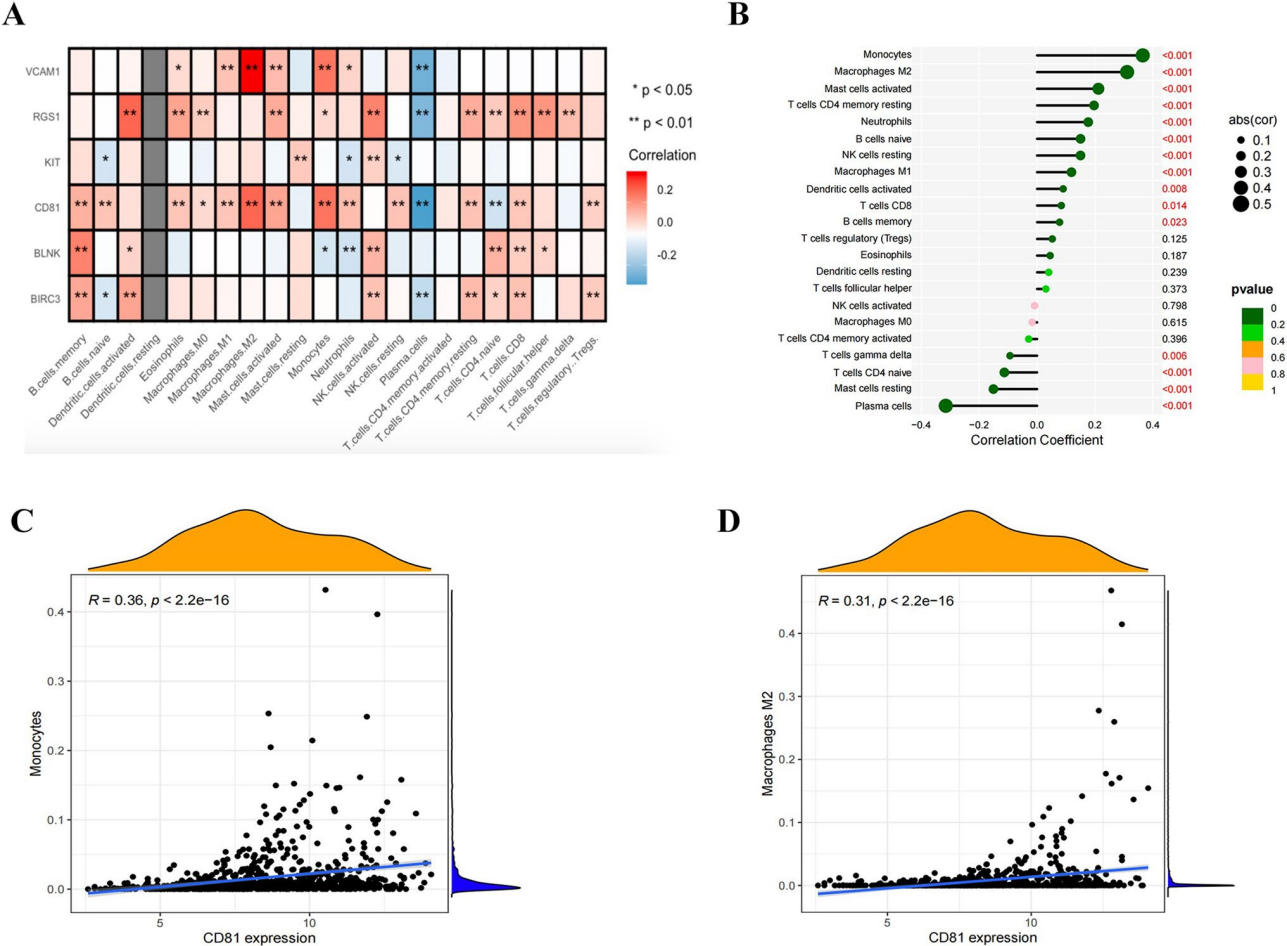
Correlation between six IRRGs risk model and differentiated immune cells. **A** Heat map of the correlation between each signature and 22 immune cell. *P < 0.05, **P < 0.01. **B** The correlation between CD81 expression and 22 types of immune cells. **C** Correlation analysis between CD81 and monocytes. **D** Correlation analysis between CD81 and macrophages M2

To further explore how prognostic IRRGs was associated with immune infiltration, we estimated the correlation between 6-gene of prognostic IRRGs and different immune cell subpopulations. The heat map shows that expression levels of RGS1 and CD81 are significantly positive correlation with most immune cell infiltration (Fig. 7A). Notably, we found that CD81 had a wide influence on the infiltration of various immune cells, especially on the infiltration of monocytes and macrophages M2. Thus, we used the lollipop diagram further to visualize the strong positive correlation between the expression of CD81 and the infiltration of monocytes and macrophages M2 (Fig. 7B). Figure 7C and D displays more details about the correlation coefficients between CD81 and these two types of immune cells.

### Verification of the prognostic value of CD81 in patients with MM by flow cytometry

**Fig. 8:**
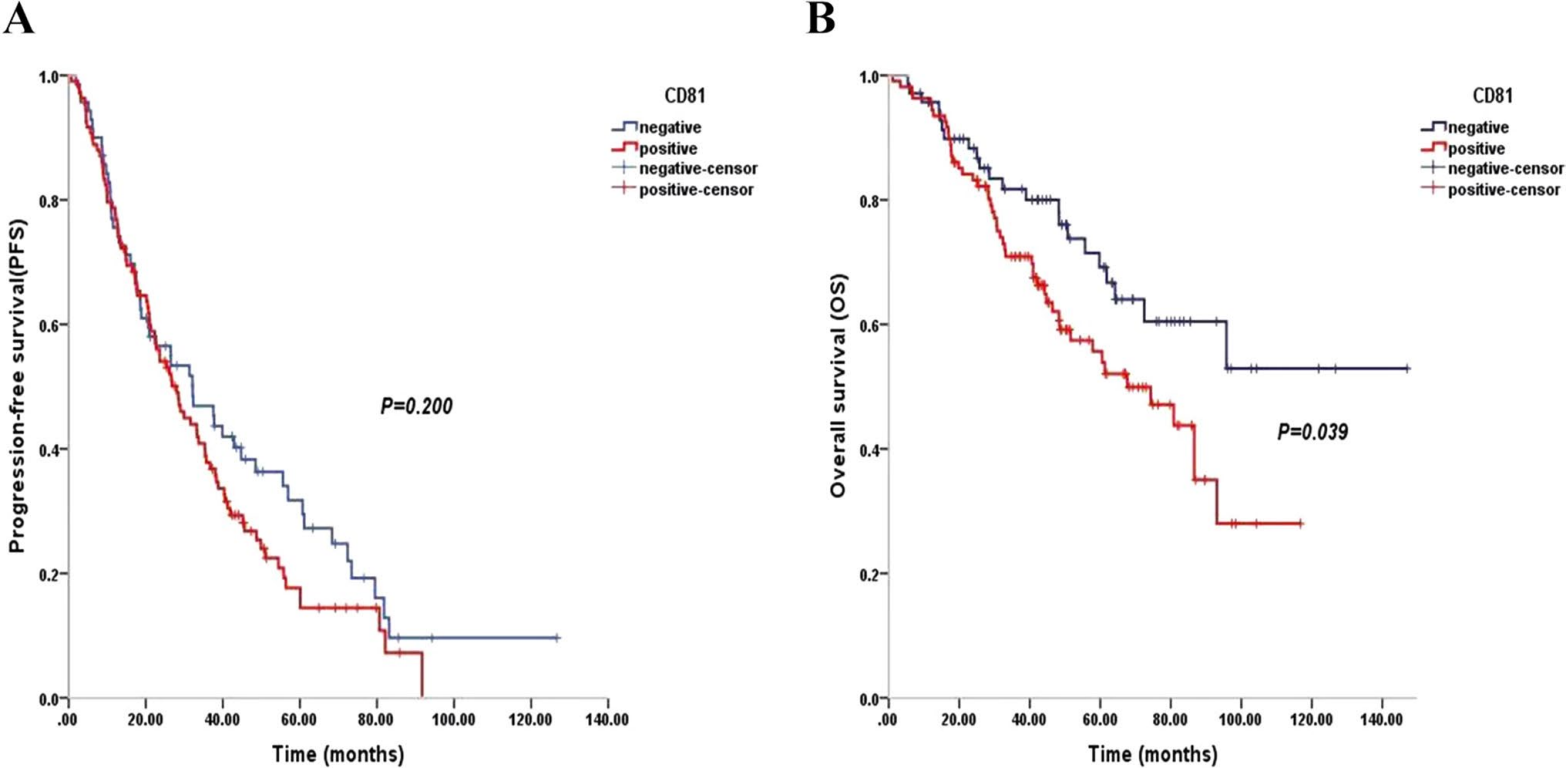
Significance of CD81 expression for outcome of MM patients. **A** Kaplan–Meier survival curves of CD81 expression for the PFS of MM patients. **B** Kaplan–Meier survival curves of CD81 expression for the OS of MM patients

To validate the different outcomes in patients with MM between CD81 positive and negative, flow cytometry was implemented to analyze the protein expression, respectively. The clinical characteristics of 178 MM patients in relation to the expression of CD81 are shown in Table 1. No correlation was found between CD81 expression and sex, age, hemoglobin, subtype, stage, cytogenetics, creatinine, serum calcium, LDH concentration, serum-free light chain (sFLC) ratio, and extramedullary infiltration. A slight increase in CD81 positive cases was observed in β2 microglobulin ≥ 5.5 mg/L group. (*P* = 0.054). The median follow-up period was 44.46 months (range, 1.10–147.07 months). In patients with CD81 positive, the median PFS was 34.48 months and the median OS was 68.06 months, compared with 43.71 months (*P* = 0.200, Fig. 8A) and 100.61 months (*P* = 0.039, Fig. 8B) for patients with CD81 negative.

## Discussion

MM is an incurable and highly heterogeneous malignant tumor, despite recent advances in therapy, approximately 20% of people newly diagnosed have substantially worse outcomes [12]. An increasing number of improved biomarkers are used for determining the overall prognosis of MM patients, but patients are still managed in a similar manner regardless of individual risk factors or disease characteristics. It means currently there is no sufficient information to routinely utilize predictive biomarkers to select initial treatment for MM or intensify treatment for high-risk MM. With the exception of tumor treatment, people pay close attention to TME. Inflammatory immune cells are an important component. Thus, we hope to explore the prognosis of MM from the perspective of inflammation and provide several evidence for the adjustment of MM therapeutic strategy.

Inflammatory response-related serum biomarkers such as interleukin-1(IL-1), sIL-2R, IL-6, IL-10 and IL-17 have a good performance in predicting prognosis of MM [13]. In addition, a bidirectional mendelian randomization study indicated that higher genetically determined monocyte-specific chemokine 3, vascular endothelial growth factor, IL-10, and IL-7 were associated with increased risk of MM [8]. However, the inflammatory response-related gene signature as prognostic marker for MM has not been reported. In our study, three MM datasets from GEO and TCGA were used to identify 24 DE-IRRGs, six of them screened out by LASSO and Cox regression for constructing a predictive model.

Our risk model consisted of VCAM1, RGS1, KIT, CD81, BLNK and BIRC3. VCAM1 is a member of the immunoglobulin super family and is produced by the expression of vascular endothelial cells, which can not only mediate inflammatory effects, but also promote angiogenesis of tumors, and lead to their growth and metastasis [14, 15]. It is also used by cancer cells to escape immune detection as its expression is up-regulated in multiple cancers, including MM, acute myeloid leukemia, where high expression associates with poor prognosis [16, 17]. RGS1, as a type of GTPase-activating protein, regulates T cell trafficking to tumors by attenuating chemokine-mediated signals and increased expression of RGS1 was associated with poorer survival of patients with breast cancer [18]. Meanwhile, reports from Korea and Egypt revealed RGS1 overexpression were associated with lower response rate and inferior OS in MM patients [19, 20]. KIT is one of the type III receptor tyrosine kinases and is involved in several signaling pathways, including the PI3K pathway, MAPK pathway, etc., responsible for cellular growth and proliferation. In medicinal chemistry and drug discovery, KIT(c-Kit) is considered one of the key targets for the management of various types of cancer, including melanoma, gastrointestinal stromal tumors, small cell lung carcinomas and acute myeloid leukemia [21]. However, relevant studies on KIT in MM are scarce. CD81 belongs to the tetraspanin family of proteins [22]. The expression of CD81 in melanoma in humans was shown to promote tumor growth and metastasis [23], and knockdown of human CD81 in osteosarcoma and breast cancer cells reduced tumor progression and dissemination [24, 25]. A previous study revealed that CD81 positive expression could potentially contribute to stratify minimal residual disease-positive MM patients after treatment and predicted inferior outcomes [26]. B cell linker protein (BLNK) is a major downstream effector of the B cell receptor signaling. On phosphorylation, it recruits multiple effectors modulating several signaling pathways, including MAPKs, ERK1/2, JNK, p38 and PLCg2 signaling, contributing to B cell development, maturation, and differentiation [27]. An experimental study has suggested that BLNK were up-regulated in Waldenström’s macroglobulinemia but not in MM, and it contributed to the regulation of Met receptor signaling in non-small cell lung cancer [28]. BIRC3 is one of the eight members of the human inhibitors of apoptosis proteins family. Many evidences point to the pro-survival and antiapoptotic role of BIRC3 in cancer cells [29]. A previous study has indicated BIRC3 inactivation is consistently associated to shorter PFS and poor OS in chronic lymphocytic leukemia patients [30]. The latest research shows the low expression of BIRC3 is correlated with poor prognosis in MM [31].

After multiple analysis and verification of multiple datasets, our model has proven patients with high-risk scores not only exhibited significantly advanced and high tumor burden, but also decreased survival rates. Therefore, this risk model could be considered as a tool to stratify patients in risk categories and it is an independent prognostic factor. Moreover, we conducted GSEA analysis based on this risk signature, and noted that the pathway in autophagy was more suppressed in up-regulated genes subgroup of the high-risk group. Autophagy mediates the delivery of various cellular cargoes to lysosomes for degradation and recycling, so that it is a quality-control, metabolic, and innate immunity process [32]. When autophagy is perturbed, this has repercussions on diseases with inflammatory components, including infections, autoimmunity and cancer. Many studies demonstrate important protective roles for autophagy against disease. However, in cancer, it seems that opposing roles of autophagy are observed in the prevention of early tumor development versus the maintenance and metabolic adaptation of established and metastasizing tumors [33]. It similarly has a dual role in the autophagy of myeloma cells. Once tumors are formed, tumor cells use autophagy to ensure their survival under nutrient-deficient and hypoxic conditions [34]. So far autophagy has been also demonstrated to be involved in the induction of MM-drug resistance [35]. Beside, we also found PI3K-Akt signaling pathway was activated and enriched in up-regulated genes subgroup, which is a conventional inflammatory signaling pathways involved in cancer development [36]. Intriguingly, enrichment scores related to GO function of cell migration and cell motility, were also elevated in up-regulated genes subgroup, suggesting that higher tumor cell activity and more potential to tumor metastasis were observed in MM patients with high-risk group. However, enrichment scores related to GO function of regulation of apoptotic process and regulation of programmed cell death were decreased. This indicated that the elimination of tumor cells might be inhibited.

A comparison of immune cells types between high-and low-risk MM groups was performed here. The results showed high-risk participants had higher proportions of B cells naïve, T cells follicular helper (Tfh), NK cells resting, monocytes and Eosinophils. These results implied that these immune cells were associated with poor prognosis. Among them, previous study revealed that the Tfh17/Tfh ratio was significantly elevated in newly diagnosed patients and even higher in relapsed patients with MM. In addition, the Tfh17/Tfh ratio was reduced in post-ASCT patients compared to that in non-ASCT patients [37]. Bone marrow monocytes are primarily committed to osteoclast formation and in the MM condition exhibit dysfunction status [38, 39]. As to Eosinophils, a study indicated that microbiota-driven interleukin-17- producing cells and eosinophils synergized to accelerate multiple myeloma progression [40].

In the study, correlation analysis between each signature and 22 immune cells showed that CD81 had a wide influence on the infiltration of various immune cells, especially on the infiltration of monocytes and macrophages M2. Our study showed that initial MM patients with CD81 positive expression by flow cytometry had poorer OS (68.06 months *vs.* 100.61 months). However, the role of CD81 in MM has not been elucidated. Furthermore, macrophages M2 can be polarized from ordinary macrophages and it can support cancer progression by several mechanisms including immune suppression, growth factor production, promotion of angiogenesis and tissue remodeling [41]. Several clinical studies confirmed macrophages M2 were associated with increased microvessel density, chemoresistance and reduced survival, independently of the MM stage [42]. A recent study pointed out that JNK signaling pathway may be involved in the growth suppression mediated by CD81 overexpression in hepatocellular carcinoma can cell [43]. Meanwhile, preclinical experiments have indicated upregulating expression of JNK and its downstream transcription c-Myc may induce macrophages toward an M2 phenotype [44]. But currently, it is unclear whether CD81 affects the prognosis of MM by promoting M2 macrophage polarization and infiltration. Thus, further study is worth to conduct in the future and CD81 might be regarded as a potential target for anti-MM therapy.

For our study, some limitations must be acknowledged. First, since MM is highly heterogeneous, some important clinical variables were not available from the public datasets. For instance, the revised-ISS stage or Mayo Stratification for Myeloma and Risk-Adapted Therapy (mSMART) risk stratification in MM patients were not assessed in our model. Therefore, greater number of clinical variables should therefore be included in future studies. Second, the relevant information of patients involved in this study was not analyzed in vitro samples. Therefore, more experiments in cell and animal model will be performed to elucidate how the gene signatures modulate the outcome of MM. Third, more independent MM cohorts should be used to validate the identified prognostic IRRGs.

## Conclusion

Our study defined and validated a novel prognostic model of IRRGs as well as other clinical features in patients with MM. The model could divide MM patients into high-risk and low-risk groups and perform well in predicting the OS of MM patients. Besides, enrichment analysis revealed adverse prognosis in high-risk patients might be highly correlated with autophagy and PI3K − Akt signaling pathways. To the best of our knowledge, our study is the first to reveal the impact of IRRGs on immune infiltration in MM and display positive correlation between CD81 and M2 macrophages. The results of this study may be useful in determining treatment strategies and providing new therapeutic biomarkers of MM in the future.

## Supplementary Information

Below is the link to the electronic supplementary material.

Supplementary file1 (CSV 12 KB)

Supplementary file2 (CSV 3 KB)

Supplementary file3 (CSV 2 KB)

Supplementary file4 (CSV 1 KB)

Supplementary file5 (CSV 0 KB)

## Data Availability

The data that support the findings of this study are all provided within the body of the manuscript.
